# Does it still fit? – Adapting affordance judgments to altered body properties in young and older adults

**DOI:** 10.1371/journal.pone.0226729

**Published:** 2019-12-30

**Authors:** Lisa Finkel, Katharina Schmidt, Jean Patrick Philippe Scheib, Jennifer Randerath

**Affiliations:** 1 Department of Psychology, University of Konstanz, Konstanz, Germany; 2 Lurija Institute for Rehabilitation Science and Health Research, Kliniken Schmieder, Allensbach, Germany; Birkbeck University of London, UNITED KINGDOM

## Abstract

Actor-related affordance judgments are decisions about potential actions that arise from environmental as well as bodily and cognitive conditions. The system can be challenged by sudden changes to otherwise rather stable actor references e.g. due to accidental bodily injuries or due to brain damage and resulting motor and cognitive constraints. The current study investigated adaptation to suddenly artificially altered body properties and its reversibility in healthy young versus older adults. Participants were asked to judge whether they would be able to fit their hand through a given horizontal opening (Aperture Task). Body alterations were induced by equipping participants with one hand splint for 24 hours that enlarged the hand in width and height. Participants were tested before and directly after putting the splint on as well as after a habituation period of 24 hours. To assess reversibility, participants were tested again directly after removing the splint and one day later. Judgment accuracy values and detection theory measures were reported. Both, young and older adults judged more conservatively when body properties were altered compared to initial judgments for normal body properties. Especially older adults showed major difficulties in such quick adaptation. Older adults’ judgment accuracy as well as perceptual sensitivity were significantly lowered when body properties were suddenly altered. Importantly, lowered judgment performance occurred for both, the splinted as well as the non-splinted hand in older adults. Only after 24 hours of habituation, older adults tended to regain initial performance levels showing adaptive behavior to the altered condition. Removing the hand splint for one day was sufficient to reverse these adaptive effects. Our study results suggest that aging slows down adaptation to sudden bodily alterations affecting actor-related affordance judgments. We propose that these altered processes may go along with uncertainty and a heightened concern about potential consequences of misjudgments. Clearly, future studies are needed to further elucidate the underlying processes of adaptation in affordance judgments. These may reveal major implications for the aging society and its associated problems with an increased risk of falling or stroke related bodily constraints.

## Introduction

The environment constantly affords actions to the observer. For example, surfaces and objects can offer opportunities or impose constraints for certain actions. Perceiving action opportunities is also termed *affordance perception* [1]. However, Gibson also stated that “affordances are properties taken with reference to the observer” [2, p. 143]. Accordingly, action opportunities are unique to the individual, meaning that they are actor-related: while the environment might afford a feasible action for one person, the same environment might not afford a feasible action for another (e.g. stepping over a 40cm high obstacle might constitute an action for an adult but not an infant). Thus, whilst perceiving affordances and judging whether an action is possible or not, our own body provides a constraining reference. It is assumed that affordance judgments are based on the interplay of on-line perceived environmental and actual body properties with a stable-built judgment criterion that has been developed based on our previous experiences [3]. A complicating fact is that the properties of our body do not remain stable throughout the lifespan. Changes are introduced by many sources, such as advancing age, limb injuries (e.g. fractures that necessitate a bandage or plaster cast) or brain damage (e.g. stroke, traumatic brain injuries) and resulting altered body properties and motor deficits (e.g. hemiplegia or hemiparesis). When our body changes, whether gradually with age or suddenly as a result of injury, it becomes necessary to adapt our affordance judgments accordingly. This presumably requires the adjustment of a learned judgment criterion.

### Adaptation to age-related bodily changes

The existing research on age-related effects in affordance judgments demonstrated ambiguous results with respect to the ability of judging action capabilities in an appropriate manner. In some studies older adults’ perceptual judgments matched their actual action capabilities reasonably well, for example when judging the maximal reach while standing and leaning forward [4] or when deciding upon the largest possible riser height for stair climbing [5, 6]. Conversely, findings from studies investigating judgements on street crossing capabilities revealed that older adults selected insufficiently large gaps in oncoming traffic [7] and underestimated the time that is required to safely cross the street [8]. In contrast, other studies reported that older adults tended to underestimate their abilities. For example, rather conservative judgment tendencies were reported in older adults when they were required to judge whether they were able to walk through doorways [9] or to inch along ledges [10]. Thus the pattern of judgment tendencies seems to be differential and heterogeneous. One could consider the various methodological approaches (e.g. task type, stimulus presentation) to investigate affordance judgments as a possible explanation for the heterogeneous pattern towards rather liberal or conservative tendencies.

However, it seems as if in elderly subjects, tendencies are more pronounced, meaning that they apply a more extreme criterion for their judgments. For example, our previous study that examined the ability to judge whether the hand fits through a given opening in young and older adults (Aperture Task; [3]) also revealed more conservative judgment tendencies in older adults. Interestingly, even when younger and older adults differed regarding their applied judgment tendency, they performed equally well when discriminating a fit from a non-fit (perceptual sensitivity). Considering the changes in judgment tendencies with aging, it seems essential for older adults to be sensitive to potential changes especially since older age also means operating within a smaller range of action capabilities. Importantly, misjudgements may potentially lead to either unnecessary avoidance behavior or they may lead to more risky behaviour that may have serious consequences (e.g. hip fractures) [5].

### Adaptation to sudden bodily changes

Prompt adaptation of affordance judgments becomes particularly necessary after sudden bodily changes due to injuries or brain damage, for which the likelihood increases with higher age [11, 12].

Evidence regarding the adaptation to sudden changes is provided by studies that examined younger adults’ ability to quickly adapt to artificially created bodily changes. Body properties were usually experimentally manipulated by enlarging body height (e.g. wearing plato-shoes [13] or heels under one’s feet [14]), body width (e.g. holding a bar, wearing backpack [15] or shoulder pads [16]) or hand width (e.g. wearing a hand prothesis [17]), by extending arm span (e.g. holding a rod in hand that prolongs arm length [18, 19]) or by increasing body weight (e.g. wearing weights [20]). Albeit there are exceptions, the majority of studies demonstrated that younger adults are able to adapt their affordance judgments to sudden bodily changes. For example, there are some studies in healthy young adults that demonstrated that even within a few trials, subjects improved in their judgments about being able to sit down with artificially altered body constraints (i.e. altered body height by wearing blocks underneath one’s feet) [21–23]. Other studies in young adults further revealed that practice interventions can be effective [15, 24, 25].

Unfortunately, all those studies that evaluated the ability to adapt to sudden changes only included young adults. To our knowledge, studies investigating the adaptation ability of elderly subjects are lacking. Therefore, we are currently unable to make any specific statements regarding older adults’ ability to adapt their affordance judgments to suddenly altered bodily constraints. And yet, it is highly likely that the adaptation process is affected by age-related cognitive and bodily decline. For example, there is ample evidence that older adults demonstrate reduced flexibility in motor capability and information processing and related neural decay [26–28]. Beyond that, studies examining whether subjects may improve their judgments after a prolonged or repeated adaptation phase are scarce (with prior experience wearing helmets (7h/week): [14], e.g. after a 8-day training period: [29]). Thus, we are currently unable to make any specific statements regarding older adults’ ability to adapt their affordance judgments to suddenly altered bodily constraints. Furthermore, the question arises, how well young and older adults may adapt their judgments when provided with a prolonged adaptation phase while being exposed to bodily alterations, e.g. for 24 hours.

On this basis, we examined young and older adults’ ability to adapt their affordance judgments to artificially altered body constraints in an Aperture paradigm including an Aperture Task requiring judgments on whether the hand fits into an opening as well as a control task examining size estimation. The chosen affordance paradigm is based on Randerath and Frey [30], who have adapted their Aperture Task from the hand-fitting task originally introduced by Ishak et al. [17].

In order to measure the ability to adapt to sudden bodily changes in the current study, we artificially manipulated the constraints of the hand. Participants were equipped with a hand splint enlarging the hand in width and height. Initial judgement ability was measured directly after splinting of the hand and after wearing the hand splint over a habituation period of 24 hours. Participants received no visual feedback at any time of investigation about whether the hand actually fit into the opening.

Next to accuracy values we analyzed participant’s perceptual sensitivity (discriminability index d’, i.e. the ability to discriminate between a fit and a non-fit) and the applied response tendencies (criterion c, i.e. tendency of judgments tending to be rather conservative versus liberal) based on a detection theory approach [31–33].

Based on indications from our previous work [3], we predicted that older adults generally would apply a more conservative judgment tendency compared to young adults at all time points: at the initial diagnostic session, directly after the splinting of one hand as well as after a habituation period of 24 hours. Moreover, we hypothesized that both, young and older adults, demonstrated lower accuracy as well as perceptual sensitivity values right after being equipped with the hand splint as compared to initial performance when bodily properties were normal. However, the group of young adults was expected to demonstrate better judgment abilities after sudden bodily changes compared to the group of older adults. Beyond that, we assumed that both young and older adults would benefit from the habituation period with a positive impact on accuracy values as well as perceptual sensitivity measures. Moreover, following previous results [3, 30, 34], we assumed that size estimation would correlate with performance in the Aperture Task at any time point.

## Methods

The project was conducted in accordance with the Declaration of Helsinki and approved by the ethics committee of the University of Konstanz.

### Participants

Participants included in the present study were tested between April 2016 and April 2018. Participants were recruited by notice board at a university setting as well as in local municipal facilities. Further, older participants were recruited via activity programs offered by the local German Red Cross as well as at retirement homes (KWA Parkstift Rosenau and Spitalstiftung Konstanz). Based on the predetermined inclusion criteria, all participants were right-handed (diagnosed with a lateralized quotient ≥ 60; [35]), had normal or corrected-to-normal vision and reported no history of psychiatric or neurologic disorders. None of the participants showed an age-inappropriate condition of mild cognitive impairment or dementia (determined with age-specific test scores of the DemTect; [36–38]). Furthermore, all participants were naïve to the study’s goals and provided informed written consent. Participation in the study was financially rewarded or compensated with study credit.

In total, 36 individuals met the inclusion criteria. This study sample consisted of younger and older adult participants with either low to medium (school education, vocational training) or high level of education (university and post-graduate education). Overall, 19 younger adults between 19 and 26 years of age (*M* = 21.68 *SD* = 2.03; 13 females, 13 highly educated) and 17 older adults between 64 and 83 years of age (*M* = 75.18 *SD* = 4.64; 8 females, 9 highly educated) were included in the present study.

Please note that part of the sample’s initial diagnostic data (beginning of session 1) has been reported as part of a different project in a previous publication [3]. Only data from initial baseline diagnostics overlapped for both studies. Subsequently, the sample was separated to investigate different questions with different experimental manipulations. While the previous study examined general affordance judgment abilities of young and older adults in the Aperture Task and its trainability (see study 1, [3]), the current study targets at examining adaptation processes to altered body properties (subjects wearing a hand splint).

### Material

**Fig 1:**
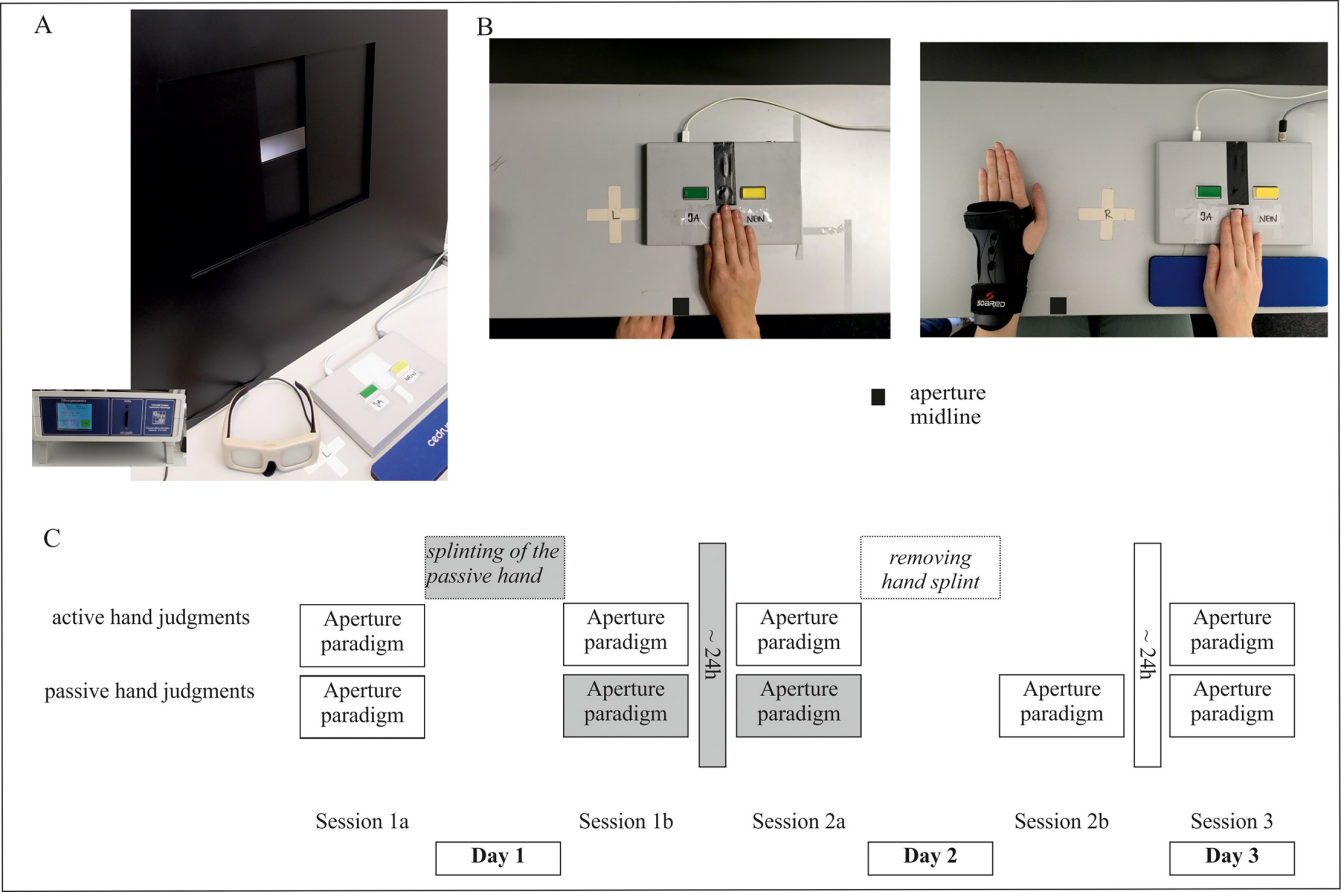
Experimental setting and procedure. (A) Experimental setting including Aperture apparatus, Plato-goggles (here: opaque), Cedrus button-pad as well as the apparatus control device. (B) The example depicts judgments for a person with an active right hand for button pressing and a passive left hand being equipped with the hand splint. The left image shows the button-pad being positioned visible on top of the stimulus mark for active right-hand judgments (hidden cross with “R”; referring to right hand as stimulus to judge). The right image shows the experimental setting for passive hand judgments (here: left splinted hand). For passive hand judgments, the button-pad with the other button-pressing hand (active), moves toward the outer edge of the table to allow the participant to position the splinted left hand visible on top of its stimulus-mark (hidden cross with “L”; referring to left hand as stimulus to judge). This shift becomes obvious from the distance to the midline (black rectangular mark). Vice versa, the positioning of the button-pad is mirrored along the aperture midline for active left-hand judgments and passive right-hand judgments. Diagram C delivers an overview of the study procedure from day 1 to day 3. On day 1 in session 1a, participant’s initial judgment performance was tested while no hand was splinted. In the subsequent session 1b, immediate effects of splinting and the ability to suddenly adapt to bodily alterations was measured. In order to investigate whether participants can improve after a 24h habituation time, judgment performance was tested again on day 2 in session 2a. Afterwards, the hand splint was removed, and we tested in session 2b whether there is an immediate effect of splint removal. Session 3 (day 3) served as control measure of reversibility. The shading of boxes indicates whether the hand to be judged was splinted (grey) or not splinted (white) at the time. (Please note that images have been partly taken from Finkel et al. [3]).

Participants took part in the experimental affordance judgment task (Aperture Task) and the control size estimation task. Both tasks used an aperture apparatus made of PVC (black board: 1000mm length x 850mm height) and aluminum profiles (framework) built by the scientific workshops at the University of Konstanz. The experimental setting including the aperture apparatus is depicted in Fig 1. The rectangular opening of the apparatus was centrally placed at the individual’s eye level and was manually adjusted in size for measuring the individual’s hand widths and heights. During the experiment, a computer-controlled step motor regulated the trial protocol-related adjustments of horizontal openings (Nanotec step motor, holding torque: 21 ncm, 1,8°/step in the full-step mode). The experiment was coded with SuperLab 5 Software (Cedrus Corporation). In order to control vision, participants wore Plato-goggles (Translucent Technologies Inc.) that could be switched between transparent and opaque throughout the experiment.

To instantly alter participants’ body properties, we used the hand splint depicted in Fig 1B. The hand splint enlarged the hand up to 1.8 cm in width (0–1.8 cm) and up to 4.2 cm in height (2.1–4.2 cm).

### Task and procedures

#### Study procedure

The present study took place on three consecutive days (approximately 90 minutes a day).

Accordingly, all participants included in this study took part in three sessions. The first session included the initial diagnostic of affordance judgment performance for normal body properties (Session 1a) as well as the ability to adapt to altered body properties immediately after splinting of the hand (Session 1b). In order to become accustomed to the new body properties, participants wore the hand splint between session 1 and session 2 for approximately 24 hours. Due to the fact that opportunities to perform daily actions were limited while wearing the hand splint (e.g. showering, cleaning dishes, ball sports), participants were allowed to remove the splint for that time. However, none of the participants removed the splint for more than a total of 4 hours. On day 2, the second session started with affordance judgments for altered body properties (Session 2a) and afterwards the hand splint was removed. Then again, participants were required to judge (Session 2b). The third session on day 3 tested whether affordance judgments were reset to the initial status. During the entire study, participants did not receive any feedback about whether the hand would actually fit into the opening, and they were not allowed to actually try to fit their hand into the opening of the Aperture Apparatus. Please see Fig 1C for an overview of the study procedure.

We counterbalanced which hand was splinted (left vs. right): for half of each group the left hand and for the other half the right hand was splinted. This allowed to evaluate potential effects of hand.

#### Measurements

At the beginning of each session, maximum height and width of the participants’ flat hands (with fingers closely spaced) were measured. The vertical opening was set to the thickness of the hand (this means the palm to the back of the hand) and the horizontal opening was tightly closed around the widest part of the hand with fingers closely spaced (for normal body properties: typically at the transition of the proximal phalanges and metacarpal bones; for altered body properties, the widest part of the splinted hand slightly moved towards the fingertips). In order to avoid visual feedback during the hand measuring procedure, the participants wore Plato-goggles that were turned opaque.

#### Aperture task

In the affordance task, also called Aperture Task, participants had to judge whether they would be able to fit their flat hand (either for normal or altered body properties) through a given horizontal opening. The presented horizontal opening sizes varied relative to the participant’s actual hand width, measured at the beginning of the session (9 openings: −1.6, −0.8, −0.4, −0.2, ±0, +0.2, +0.4, +0.8, and +1.6cm). The ±0-opening corresponds to maximum hand width. In order to avoid an imbalance towards more frequent “yes”-trials (5 openings with ±0, +0.2, +0.4, +0.8, and +1.6cm) compared to “no”-trials (4 openings with -0.2, -0.4, -0.8, and -1.6cm), we added one filler trial per block presenting smaller openings for which the correct answer would be “no” as well (openings with either -2.0, -3.0 or -4.0.cm). Please note that the negative filler trials are much larger than the 0-trials that reflected actual hand size which is why correct responses are likely (i.e. less errors on false alarms). Filler trials were excluded for the later analysis. Due to the fact that a previous study [30] indicated that a stable judgment tendency seemed to be formed during the first few judgment trials, we added a familiarization block of 20 trials in the first initial diagnostic session before the experimental blocks (32 trials including 3x9 openings, 3 filler trials and 2 extreme openings). In the other sessions, we only added 6 introductory trials in order to accustom participants to the task, and to ensure that task instructions were understood. Judgments for the left or the right hand were blocked. Since use of the splinted hand was restricted, button-pressing was delegated to the non-splinted hand (i.e. right hand was assigned to be the “active” hand for participants with left splinted hand and vice versa). This goes along with previous applications of this paradigm in stroke patients who frequently are constrained by hemiparesis. Since in session 1a, 2b and 3 no hand was splinted, we used the terms “active” for the button-pressing hand and “passive” for the hand that did not press buttons. The passive hand was designated to be splinted and was tested with splint in sessions 1b and 2a. When describing behaviour in these 2 sessions we added the words “splinted” (passive splinted) and “non-splinted” (active non-splinted) to classify the hands.

Sessions with judgment blocks for the right-hand active group (left hand splinted) were presented in the order: right, left, right, left (RLRL) and LRLR for the left-hand active group (right hand splinted). In all blocks, judgments were indicated by either pressing “yes” or “no” on a button-pad (Cedrus RB540). The hand to be judged was positioned on a stimulus-mark, shifted 8 cm to the left or right of the aperture’s vertical midline (see Fig 1B right). This prevented participants from employing direct alignment strategies. In blocks of judging the active hand, the button-pad with the active hand was set on the top of the stimulus-mark (Fig 1B left). In passive hand judgments, the pad with the active hand was moved 20cm towards the external lateral edge of the table and the passive hand was positioned on the respective stimulus-mark (Fig 1B right). Please note that for time reasons we refrained from adding another active hand judgment block at session 2b.

#### Size estimation task

In this control task, we assessed horizontal size estimation ability. Thus, this task specifically measures the participants’ visual-perceptive abilities, whereas movement planning and the integration of bodily and environmental information is not necessarily required to accurately solve the task. Participants were instructed to verbally indicate when a gradually adjusted opening had the same size as the widest part of the hand (depending on stage of investigation either for normal or altered body properties). The hand to be judged was positioned visible in front of the participant. Participants were allowed to correct their judgments until satisfied with the final opening size. The vertical opening was kept constant corresponding to the actual vertical height of the hand to be judged. The horizontal opening was either gradually decreased starting from 20cm or increased starting from a closed opening (0cm). Increasing and decreasing trials were presented in alternation and judgments for left and right hand were presented in a fixed randomized order.

### Data analysis

Behavioral data were analyzed with SPSS Statistics 25 (IBM). Statistical significance was determined by reporting p-values two-tailed (*p* < .05) and, whenever computing power was sufficient, exact instead of asymptotic. Behavioral data were analyzed non-parametrically, because Shapiro-Wilk Test and screening of normal probability plots indicated that part of the data was not normally distributed in both, young (accuracy: active hand, sessions 1a, 1b and 2; perceptual sensitivity and criterion: active hand, session 3; *W*(19) ≥ .866. p ≤ .046) and older adults (accuracy: passive hand, session 1b; perceptual sensitivity: active hand, sessions 1b and 3; criterion: active hand: session 3; passive hand, sessions 1b, 2a and 3; *W*(17) ≥ .818. p ≤ .035).

#### Dependent variables determining affordance judgment behavior

We analyzed general judgment accuracy (percent of correct judgments) and two independent detection theory variables: perceptual sensitivity (discriminability index d-prime) and judgment tendency (criterion c) [31–33]. Here we do not calculate ratios (judged / actual boundaries) since this absolute measure appears disadvantageous for the current methodological approach presenting fixed openings with unequal intervals (0 +/- 2, 4, 8, 16mm) as opposed to continuous stimulus presentation (i.e. gradually opening/closing the aperture).

The discriminability index measures the ability to perceptually discriminate a fit from a non-fit. The higher the d-prime value, the better the perceptual discrimination performance. The criterion c describes a participants’ judgment tendency. While negative criterion values are associated with rather liberal judgment tendencies (i.e. respond “yes” more often than the ideal observer), positive criterion values represent more conservative judgment tendencies (i.e. respond “no” more often than the ideal observer). Calculation of these two detection theory variables were based on Hit and False-Alarm rates.

Thus, the consideration of d-prime as a perceptual measure and criterion c as a measure of conservative versus liberal judgment tendencies allowed a more precise interpretation of participants’ judgment behavior, considering the type of error that had been conducted: False-Alarms (indicating “yes”-answers even when the hand does not fit through the given aperture) versus missed Hits (indicating “no”-answers while the hand would actually fit through the aperture). Whereas the False-Alarm rate depicts the ratio of False Alarms and the total number of actual negative events, the Hit rate depicts the ratio of the number of Hits (positive events successfully categorized as positive; i.e. indicating “yes” in trials the hand actually fits through the given opening) and the total number of actual positive events. The perceptual sensitivity (d-prime) and the judgment tendency (criterion c) were calculated from these rates using the following formulas: *d-prime* = Z(Hit rate)—Z(False-Alarm rate) and *c* = -.5*[Z (Hit rate)+Z(False-Alarm rate)].

Summarized, we evaluated participants’ judgment behavior by analyzing accuracy values as well as perceptual sensitivity and judgment tendency measures. Furthermore, we calculated the perceived affordance boundary: the percentage of “yes”-responses (i.e. in which participants judged the opening as wide enough to fit the hand through) for each of the 9 aperture widths was calculated using a logistic function (cf.[39, 40]). We further specified the 50% boundary, that is, the aperture size where participants changed their preference for “no”-responses (not able to fit) to “yes”-responses (able to fit) (cf [41]).

#### Within subject analyses

Based on our stated hypotheses, we ran a Friedman Test to evaluate whether there was a main effect of timepoint of measurement (initial judgments for normal body properties; judgments for altered body properties directly after hand splinting; judgments for altered body properties after a habituation period of 24 hours) within each age group and per variable (accuracy and signal detection data, separated for hand to be judged). Based on significant results, post-hoc tests (Wilcoxon Test) were run to further specify the ability to quickly adapt affordance judgment performance to altered body constraints (normal hand judgments in session 1a versus altered hand judgments in session 1b) and the effect of the habituation period on the adaptation process (altered hand judgments in session 1b versus altered hand judgments after 24h in session 2a). In order to assess whether judgments for altered body properties after a habituation period are comparable with judgements for normal body properties, we further compared normal hand judgments versus altered hand judgments after 24 hours. In addition, it was evaluated whether the experimental manipulation significantly affected judgment behavior over time, we invited the subjects again after 24 hours of removal of the splint and compared initial judgment behavior at session 1a with judgments at session 3. To correct for family-wise error rate, we adjusted *p*-values using the stepwise Holm-Bonferroni procedure (p_adj_). Wilcoxon z values were used to calculate the effect size *r* [42] by dividing z by the square root of N (the procedure is also suggested by Fritz et al. [43]).

Please note, to limit the number of executed comparisons, we did not run pairwise comparisons results for session 2b, in which participants judged the affordances for their passive hand for normal body properties directly after removing the hand splint, in the results. However, for the sake of completeness, variable values were nevertheless plotted in the respective figures.

#### Correlations with size estimation

Size estimation ability has been separately collected and evaluated per session (normal body properties in session 1a, altered body properties in session 1b and altered body properties after 24h in session 2a). To analyze correlations between affordance judgment performance and size estimation values, Kendall's tau was applied.

## Results

Affordance judgment behavior was analyzed by considering accuracy and signal detection values as dependent variables. Data of the dependent variables (accuracy, perceptual sensitivity, criterion) was not significantly affected by the side of hand that was splinted [between subjects variable: splinting the right hand (with the left hand pressing buttons) vs. splinting the left hand (with the right hand pressing buttons): *U* ≥ 104.5, p ≥ .071].

Between subjects comparisons demonstrated that young and older adults did not differ in accuracy and perceptual sensitivity but applied different judgment tendencies (accuracy: U ≥ 109.0, p ≥ .095; perceptual sensitivity: U ≥ 118.0, p ≥ .172; judgment tendency: U ≥ 48.5, p ≤ .001). Please note, a similar statement was written in our previous study with partly overlapping subjects [3]. In order to detect effects of adaptation per age group, we compared performance over time for percentage of accurate responses, d-prime and criterion values. Data was analyzed separately per respective hand that had to be judged (active/non-altered or passive/altered).

In a first step, we analyzed whether there was a main effect of time (3 time-points: initial performance with normal body properties vs. directly after splinting vs. after wearing splint for 24 hours). Post-hoc, the participants’ ability to adapt to sudden bodily changes was evaluated (initial performance vs. directly after splinting), and potential effects of habituation were identified (directly after splinting vs. after wearing splint for 24 hours and initial performance vs. after wearing splint for 24 hours).

**Fig 2:**
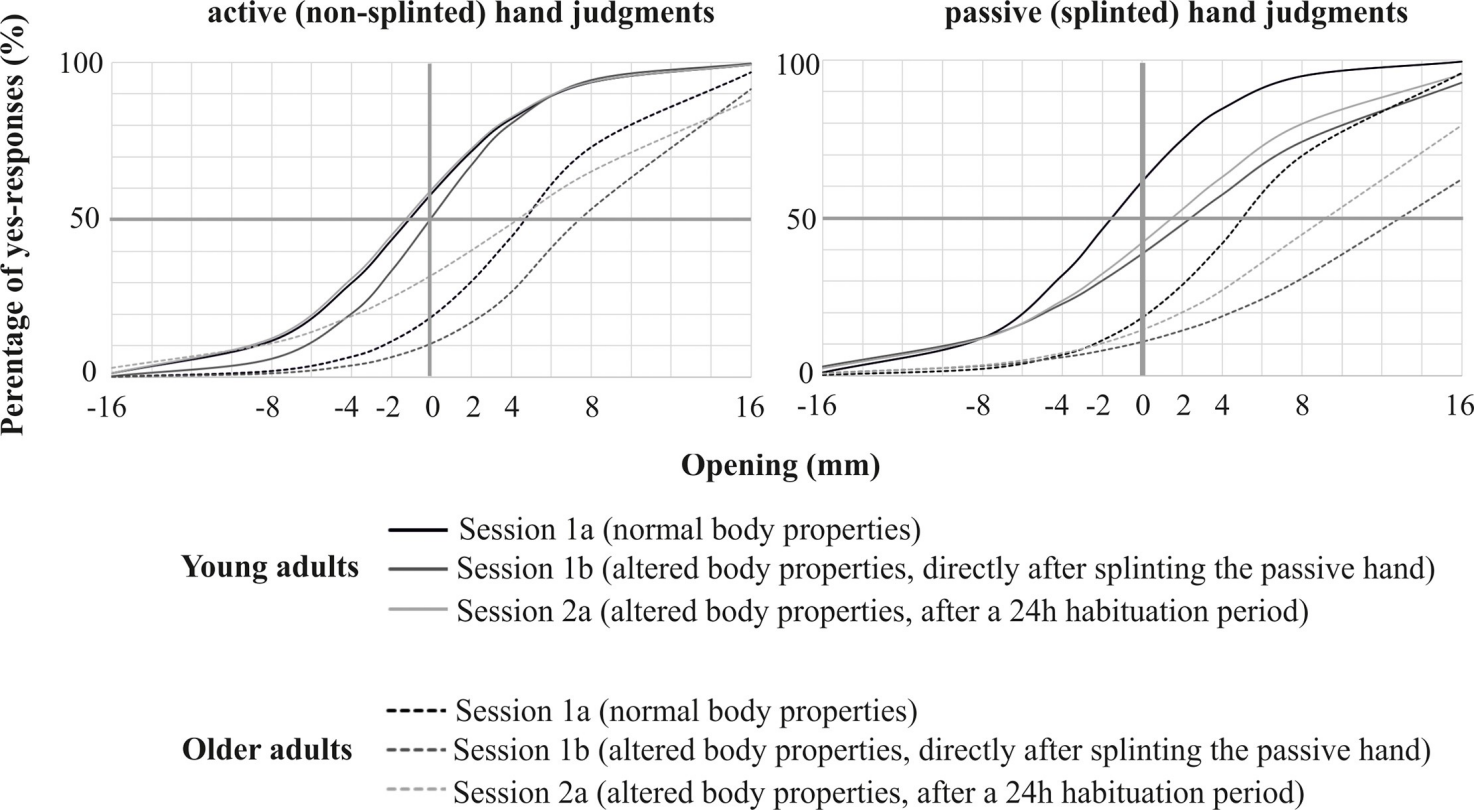
Logistic fits on proportions of yes-responses for passive hand judgments. The graph depicts the fitted proportions for both age groups and sessions as a function of aperture width. The point where the trend lines cross the horizontal grey line indicates the 50% perceived action boundary. Data below the line indicate a preference for “not able to fit”, whereas data above the line indicate preference for “able to fit”.

**Fig 3:**
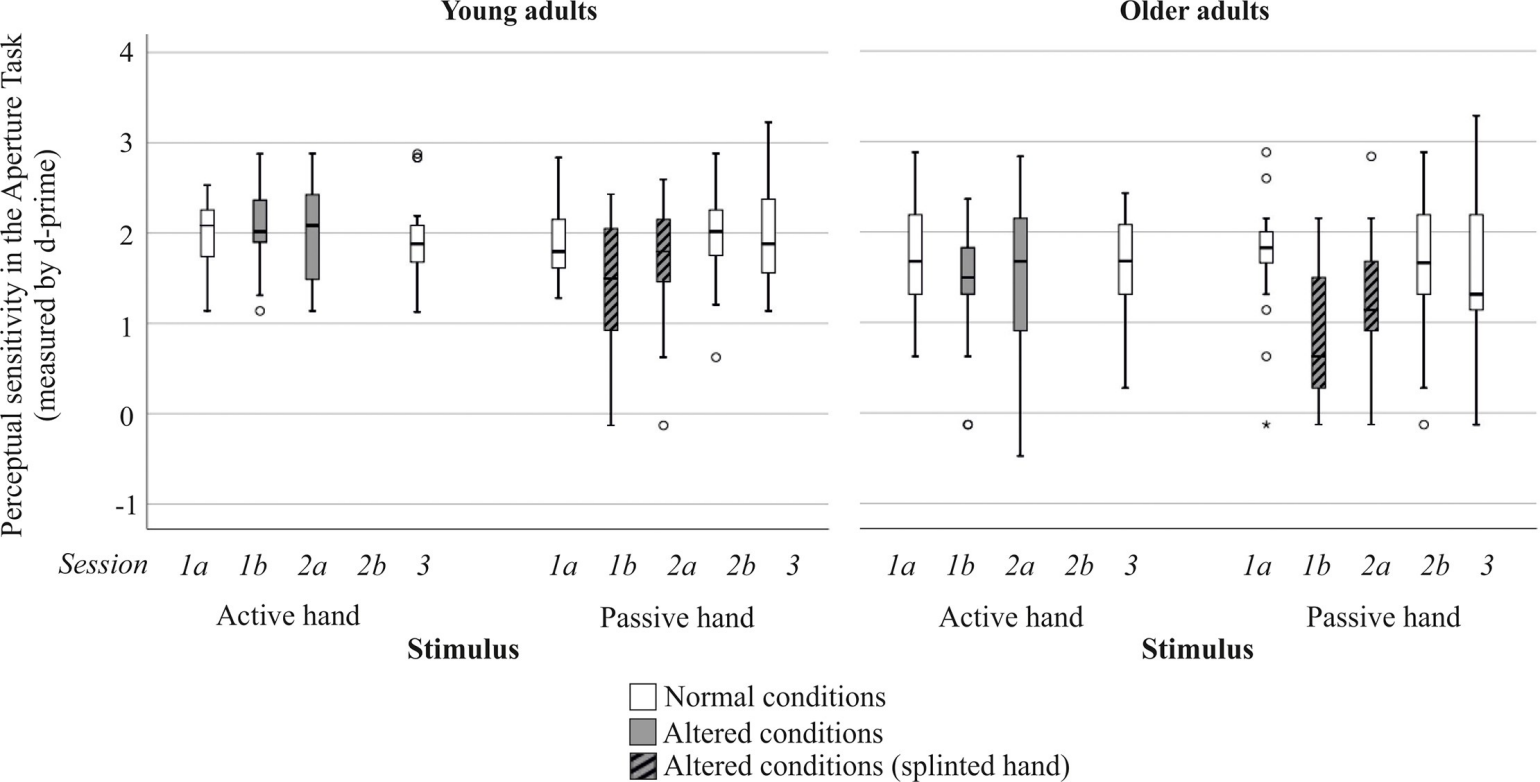
Perceptual sensitivity measured by d-prime separated by hand to be judged and session. Boxplots in grey refer to sessions where body properties were altered. Grey boxplots with black stripes indicate the hand that has been splinted. Please note that participants were only required to judge affordances for their passive hand in session 2b.

**Fig 4:**
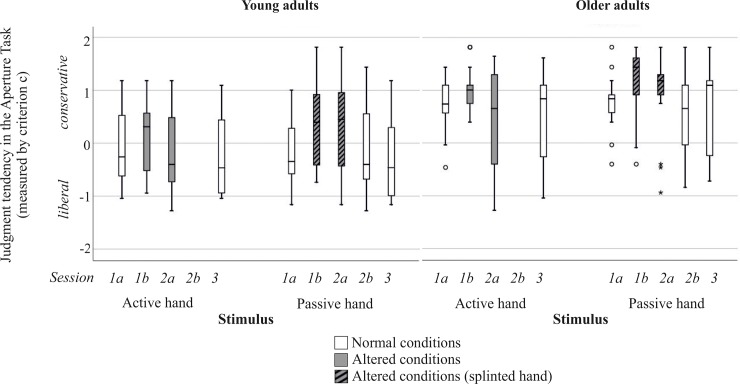
Judgment tendency measured by criterion c separated for hand to be judged as well as sessions. Grey boxplots refer to sessions with altered body properties. Grey boxplots with black stripes indicate judgments for a splinted hand. Please note that participants were only required to judge affordances for their passive hand in session 2b.

**Fig 5:**
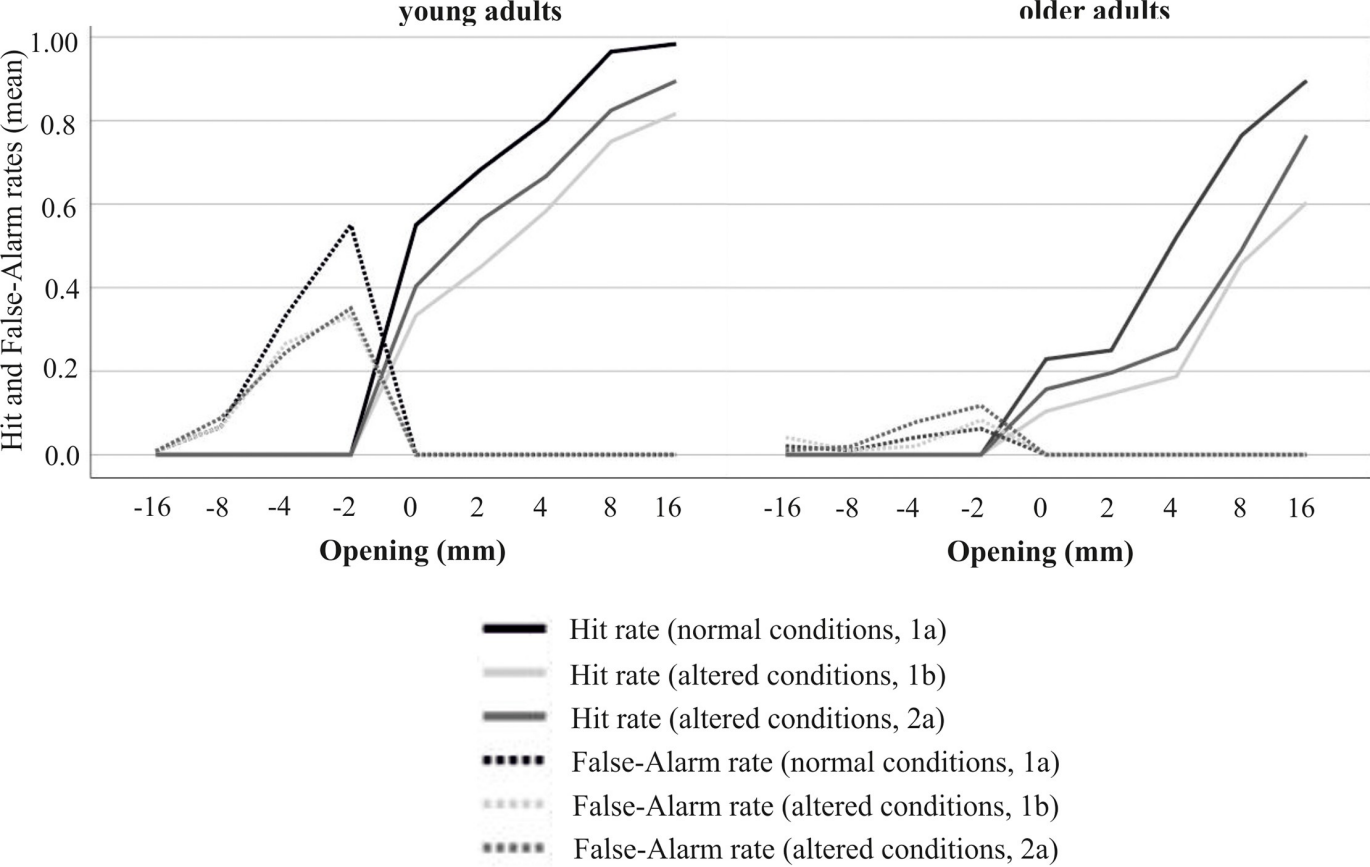
Hit and False-Alarm rates for passive hand judgments separated for age groups. Graphs show the distribution of Hit and False-Alarm rates over openings. In order to display the adaptation processes, one line per session was depicted. (1a = normal body properties, 1b = altered body properties directly after splinting of the hand, 2a = altered body properties after 24h of wearing the hand splint).

Results are shown in Figs 2 to 5. Descriptive data are detailed in Table 1. Post-hoc Wilcoxon Test results are listed in Table 2. Below, we only report pairwise comparisons for variables that revealed a significant main effect of time.

**Table 1:** Descriptive data for young and older adults.

			Initial performance with normal body properties (1a)	Directly after splinting (1b)	After wearing splint for 24 hours (2a)	Control measure with normal body properties (3)
	Variable	Hand	*Mdn* *[IQR]*	*Mdn* *[IQR]*	*Mdn* *[IQR]*	*Mdn* *[IQR]*
Young (N = 19)	Acc (%)	A	81.48 [74.07, 85.19]	81.48 [77.79, 85.19]	81.45 [70.37, 88.89]	77.78 [74.07, 88.89]
		P	77.78 [74.07, 85.19]	74.07 [59.26,81.48]	77.78 [66.67, 85.19]	77.78 [74.07, 88.89]
	d-prime	A	2.08 [1.68, 2.32]	2.02 [1.88, 2.36]	2.08 [1.48, 2.53]	1.88 [1.68, 2.15]
		P	1.80 [1.57, 2.15]	1.49 [0.92, 2.08]	1.80 [1.44, 2.15]	1.88 [1.44, 2.43]
	Criterion c	A	-0.26 [-0.72, 0.66]	0.31 [-0.52, 0.57]	-0.40 [-0.74, 0.66]	-0.46 [-0.94, 0.48]
		P	-0.34 [-0.64, 0.28]	0.40 [-0.56, 1.00]	0.45 [-0.46, 1.00]	-0.46 [-1.04, 0.45]
Older (N = 17)	Acc (%)	A	70.37 [59.26, 83.33]	66.67 [59.26, 75.93]	70.37 [53.70, 85.19]	74.07 [57.41, 83.33]
		P	74.07 [65.95, 79.63]	51.85 [48.19, 68.52]	59.26 [51.85, 74.07]	62.96 [57.41, 85.19]
	d-prime	A	1.68 [1.11, 2.28]	1.50 [1.11, 1.84]	1.67 [0.77, 2.15]	1.68 [0.97, 2.08]
		P	1.83 [1.48, 2.01]	0.62 [0.27, 1.59]	1.14 [0.59, 1.78]	1.31 [1.02, 2.22]
	Criterion c	A	0.74 [0.37, 1,20]	1.00 [0.74, 1.20]	0.66 [-0.40, 1.37]	0.84 [-0.49, 1.27]
		P	0.84 [0.52, 1.01]	1.44 [0.87, 1.61]	1.18 [0.83, 1.45]	1.10 [-0.32, 1.24]

*Note*. Mdn = median, IQR = interquartile range, A = active button-pressing hand to be judged, P = passive hand (splinted in: sessions 1a, 2b) to be judged

**Table 2:** Post-hoc analyses determining young and older adults’ ability to adapt to sudden bodily changes (session 1a versus session 1b), potential effects of habituation (session 1b versus session 2a), comparability with initial performance (session 1a versus session 2a) as well as control measure of reversibility (session 1a versus session 3). (Wilcoxon-Tests).

			Effects of sudden change 1a versus 1b				Effects of habituation 2a versus 1b				24hours splint-wearing compared to initial performance 2a versus 1a				Control (reversibility) 3 versus 1a			
	Variable	Hand	*Z*	*p*	*p*_*adj*_	*r*	*Z*	*p*	*p*_*adj*_	*r*	*Z*	*p*	*p*_*adj*._	*r*	*Z*	*p*	*p*_*adj*_	*r*
Young (N = 19)	Acc (%)	A	-	-	-	-	-	-	-	-	-	-	-	-	-0.97	.350	-	-
		P	*-2*.*45*	.*013*	-	.*397*	-	-	-	-	-	-	-	-	-0.13	.907	-	-
	d-prime	A	-	-	-	-	-	-	-	-	-	-	-	-	-0.64	.548	-	-
		P	*-3*.*12*	.*031*	-	.*506*	-	-	-	-	-	-	-	-	-0.44	.672	-	-
	Criterion c	A	-	-	-	-	-	-	-	-	-	-	-	-	-0.54	.611	-	-
		P	-2.72	**.005**	**.020**	.441	-0.68	.523	>.999	.110	-1.76	.081	.243	.286	-0.56	.588	.558	.091
Older (N = 17)	Acc (%)	A	-2.58	**.006**	**.024**	.442	-1.10	.292	.876	.189	-0.52	.626	>.999	.089	-0.51	.627	.627	.087
		P	-2.73	**.004**	**.020**	.468	-1.37	.184	.368	.235	-2.19	**.026**	.078	.376	-0.86	.411	.411	.147
	d-prime	A	*-2*.*49*	.*011*	-	.*427*	-	-	-	-	-	-	-	-	-0.92	.371	-	.158
		P	-3.12	**.001**	**.004**	.535	-1.73	.086	.172	.397	-2.36	**.016**	**.048**	.405	-0.76	.466	.466	.130
	Criterion c	A	*-2*.*68*	.*005*	-	.*460*	*-2*.*33*	.*017*	-	.*340*	-	-	-	-	-1.49	.142	-	.256
		P	-2.56	**.008**	.**032**	.439	-1.79	.075	.225	.307	-1.68	.097	.194	.288	-0.66	.525	.525	.113

*Note*. Significant post-hoc comparisons are bold printed. Further, significant Wilcoxon results for variables with no significant effect of session (indicated by Friedman) are written italic. As an ethical issue, control comparisons of recovery were reported regardless of whether Friedman results reached significance or not. Effects of r can be interpreted based on Cohen [42] with the following intervals: r: .1 to .3: small effect; .3 to .5: intermediate effect; .5 and higher: strong effect. A = active button-pressing hand to be judged, P = passive hand (splinted in: sessions 1a, 2b) to be judged, p_adj_ = Holm-Bonferroni adjusted p-values.

### Adaptation to bodily alterations

#### Accuracy

The descriptive data of accuracy values per group and session are provided in Table 1. Friedman Test results revealed a main effect of time of measurement (normal body properties, altered body properties, altered body propertied after 24h) for judgment accuracy in older adults (active hand: χ^2^(2) = 7.355, *p* = .024; passive hand: χ^2^(2) = 11.934, *p* = .002). However, there was no significant main effect of time of measurement in young adults (active hand: χ^2^(2) = 0.114, *p* = .951; passive hand: χ^2^(2) = 4.394, *p* = .114).

Table 2 provides data on the following post-hoc comparisons. Since the Friedmann Test results revealed no significant effect of time of measurement for younger adults (*p* ≥ .114), we refrained from further pairwise comparisons. Post-hoc comparisons in older adults demonstrated, that judgments for suddenly altered body properties (in session 1b) were less accurate than judgments for normal body properties (in session 1a). Interestingly, accuracy values were not only significantly lower for judgments concerning the splinted hand but also concerning the non-splinted hand in session 1b. Descriptive statistics indicated that judgment accuracy values for the splinted hand tended to improve after a 24 hours habituation period (see medians in Table 1). However, this effect did not reach statistical significance. After 24 hours, accuracy values for the splinted hand were still significantly lower compared to the initial judgment accuracy for normal body properties. The deviating effect for the non-splinted hand from the initial judgment accuracy was no longer apparent at that point in time.

As a result of calculating the perceived affordance boundary, Fig 2 depicts the logistic fits for active (non-splinted) and passive (splinted) hand judgments before splinting, directly after splinting and after a 24h habituation time for young and older adults. We further specified the critical 50% point. With normal body properties, younger adults deviate -1.12 mm for the active, button-pressing hand and -1.54 mm for the passive hand, whereas older adults deviate 4.72 mm in active hand judgments and 5.14 mm when judging their passive hand. Directly after splinting the hand, the deviations from actual hand sizes (actual hand size = 0) were the following: young adults: -0.07 mm (active button-pressing hand), 2.44 mm (passive splinted hand); older adults: 7.52 mm (active button-pressing hand), 12.95 mm (passive, splinted hand). After 24h splinting, the deviations from actual hand size were reduced: young adults: -1.33 mm (active button-pressing hand), 1.50 mm (passive splinted hand); older adults: 4.31 mm (active button-pressing hand), 9.08 mm (passive, splinted hand).

#### Perceptual sensitivity

Table 1 provides descriptive data for perceptual sensitivity per group and session. Friedman Test results revealed a main effect of time of measurement (normal body properties, altered body properties, altered body properties after 24h) for perceptual sensitivity in older adults judging the passive hand (χ^2^(2) = 11.934, *p* = .002). However, there was no significant main effect of time of measurement for active hand judgments in older adults (χ^2^(2) = 4.364, *p* = .118) and for neither hand of the younger adults (active hand: χ^2^(2) = 0.243, *p* = .894; passive hand: χ^2^(2) = 3.694, *p* = .163). Thus, we here only provide pairwise comparisons for older adults’ perceptual sensitivity values when judging their passive hand (see also medians in Table 1 and boxplots in Fig 3).

These post-hoc comparisons in older adults demonstrated a significantly lower perceptual sensitivity performance in judgments for suddenly altered body properties (i.e. actually splinted hand) than for normal body properties (see Table 2). After a 24 hours habituation period perceptual sensitivity tended to improve under altered conditions. However, older adults still judged significantly worse after this habituation period (session 2a) compared to initial judgments given normal body properties (session 1a). Thus, they seemed to not fully adapt to altered bodily constraints within the given timeframe.

#### Judgment tendency

Descriptive data of judgment tendency values per group and session are provided in Table 1. Friedman Test results revealed a main effect of time of measurement (session 1a: normal body properties, session 1b: suddenly altered body properties, session 2a: altered body properties after 24h) for judgment tendency in young and older adults judging the passive hand (χ^2^(2) ≥ 10.361, *p* ≤ .004). However, neither group showed a significant main effect of time of measurement for judgment tendency in active hand judgments (χ^2^(2) ≥ 0.730, *p* ≥ .052). Thus, below we only provide pairwise comparisons for passive hand judgments. Results are shown in Fig 4 and Tables 1 and 2. Post-hoc comparisons demonstrated that young adults judged more conservatively for the passive hand when wearing the hand splint as compared to their rather liberal judgment tendency when body properties were normal. The more conservative tendency is also observable after the habituation period of approximately 24 hours. Thus, in adapting to new body constraints, young adults seemed to apply a judgment tendency that strongly deviates from the judgment tendency being applied for their normal body properties.

While young adults judged more liberal for normal body properties, older adults applied conservative judgments right from the beginning. And yet, after the passive hand was splinted, older adults’ conservative tendency was significantly enhanced. After a 24 hours habituation period older adults tended to adjust and used less conservative judgements, but their judgments were still more conservative than their initial judgments for normal body properties (session 1a), indicating rather slow adaptation.

#### Hit and False-Alarm rates

In order to facilitate the interpretation of the detection theory results, we descriptively visualized Hit and False-Alarm rates for passive hand judgments across trial types. Fig 5 illustrates how the judgment behavior of young and older adults ‘evolved’ through normal and altered conditions. When body properties were suddenly altered, younger adults less frequently responded yes (I can fit my hand into the opening) reflected by lower Hit and False-Alarm rates. After 24 hours, there is only a slight increase in Hit rates, whereas False-Alarm rates remain on the same level. Contrarily, older adults responded yes less frequently from the beginning. There were almost no False-Alarm errors in older adults, neither for normal nor for altered body properties. In addition, when body properties were altered the number of Hits was considerably reduced.

### Recovery: Performance in normal body properties before versus after the splinting manipulation (session 1a vs. session 3)

In addition, we analyzed whether participants would be able to reverse their adaptive judgement behavior. To check whether judgment behavior under normal body properties conditions was changed by the splinting-manipulation, we compared initial judgment behavior in session 1a with judgments in session 3,—after the hand splint was removed for 24 hours. Again, per group we separated for hand to be judged. Post-hoc comparison for all three variables showed no significant differences, neither for young nor for older adults (see Table 2). To illustrate this behavior, Figs 3 and 4 also display variable values of session 3 in boxplots.

#### Size estimation

In a final step, we analyzed potential effects of size estimation ability on the affordance judgment performance. By using a gradual hand task, size estimation data was collected and evaluated per session (normal body properties, altered body properties, altered body properties after 24h) and per hand (active non-splinted hand vs. passive splinted hand). For descriptive and inferential statistics see Table 3.

**Table 3:** Descriptive and inferential statistics of size estimation ability in the gradual hand task. Wilcoxon-Tests analyses determine potential effects of session (session 1a versus session 1b; session 1b versus session 2a) in young and older adults’ size estimation ability.

			Initial performance		Effects of sudden change 1a versus 1b				Effects of habituation 2a versus 1b			
			Session 1a		Session 1b				Session 2a			
	Variable	Hand	*M*	*SD*	*M*	*SD*	*Z*	*p*	*M*	*SD*	*Z*	*p*
Young (N = 19)	Mean deviation	A	1.03	0.85	0.98	0.94	-	-	1.13	0.86	-	-
		P	1.23	0.93	1.56	1.29	-1.95	.051	1.59	1.09	-0.14	.899
Older (N = 17)	Mean deviation	A	2.15	1.15	2.28	1.59	-	-	2.10	1.28	-	-
		P	1.89	1.31	2.89	1.88	-2.58	.008	2.68	1.52	-.88	.397

*Note*. A = active button-pressing hand to be judged, P = passive hand (splinted in: sessions 1a, 2b) to be judged

First, results from the size estimation task for normal body properties revealed that on average, young and older adults estimated their hands to be larger than they actually were.

Second, Friedman Test results revealed a main effect of time of measurement (session 1a: normal body properties, session 1b: suddenly altered body properties, session 2a: altered body properties after 24h) for estimating the splinted hand’s size in both age groups (χ^2^(2) ≥ 9.89, *p* ≤ .006). Neither group showed a significant main effect of time of measurement for active hand estimations (χ^2^(2) ≥ 1.44, *p* ≥ .415). Thus, we only provide pairwise comparisons for passive hand estimations. In both age groups, the overestimation of the hand size was larger directly after splinting (session 1b) compared to before (session 1a). After the habituation time of 24 hours of splinting, the size estimation ability for the splinted hand did not improve after this habituation time.

In younger adults, the mean difference between estimated and actual hand size correlated with the applied judgment tendency for the respective hand (active vs. passive) in all three sessions. For active and passive hand size estimations, the overestimation of hand size went along with more conservative judgment tendencies (for normal body properties: r(19) ≥ .408, p ≤ .016; for altered body properties: r(19) ≥ .552, p ≤ .002; after 24h: r(19) ≥ .439, p ≤ .008). For passive hand estimations, the size estimation ability was further associated with the perceptual sensitivity measures for normal body properties in session 1a (r(19) = -.337, p = .046) and accuracy values for the splinted hand in session 1b (r(19) = -.363, p = .036). In both cases, larger misestimating went along with worse performance.

In older adults, the ability to accurately estimate size was also correlated with judgment tendencies applied for the active or passive hand (for normal body properties: r(17) ≥ . 376, p ≤ .041; for altered body properties: r(17) ≥ .371, p ≤ .045; after 24h: r(17) ≥ 424, p ≤ .020).

When the passive hand was splinted, older adults’ ability to accurately estimate the passive hand’s size was associated with accuracy and perceptual sensitivity measures for this hand. Larger misestimating went along with lower judgment accuracy and perceptual sensitivity values directly after hand splinting (accuracy: r(17) = -.409 , p = .025; d-prime: r(17) = -.434, p = .018). These correlations became significant after 24h of habituation (accuracy: r(17) = -.418, p = .022, d-prime: r(17) = -.428, p = .020). Furthermore, size estimation ability was further correlated with perceptual sensitivity measures for the active hand when initially measured at session 1a and after 24h (r(17) ≥ -.452, p ≤ .038). Larger misestimating further went along with lower accuracy values in session 1a (r(17) = -.402, p = 0.30).

## Discussion

Accurate affordance judgments are based on the fit between perceived environmental properties and one’s own body capabilities. It is assumed that affordance judgments involve the perception of environmental properties as well as the processing of an experience-based judgment criterion. However, bodily capabilities can be subject to change. For instance, body constraints can change due to unfortunate consequences of injuries (e.g. wearing a bandage or plaster cast) or brain damage such as stroke (e.g. hemiplegia or hemiparesis). When body properties change, related action opportunities change concurrently. It becomes necessary to adapt affordance judgment to these changing body properties. We propose that this also includes the adjustment of a standard on which a judgment or decision may have been based, the current judgment criterion. Furthermore, since bodily and cognitive capabilities typically change with increasing age, older adults have to meet additional requirements concerning the adaptation of affordance judgments and the related judgment criterion. Thus far, there are only few studies that examined older adults’ ability to adapt to sudden bodily changes.

With the current work, we aimed at elucidating the ability to adapt to changes in body properties. We examined young as well as older adults’ judgment performance with normal and artificially altered body properties. Participants judged whether their hand could fit into an aperture. Body properties were altered by splinting either the left or the right hand for 24 hours. Participants were tested twice while wearing the splint: directly after the hand was splinted (begin of adaptation process) and after 24 hours of habituation (adaptation process after 24h practice).

In young adults, judgment tendencies but not the ability to discriminate a fit from a non-fit appeared to be affected by changing body constraints. Even though on a descriptive level, judgment performance seemed to slightly decline directly after hand splinting, after 24 hours, accuracy and perceptual sensitivity were comparable for judging the hand’s fit between conditions of normal and altered body properties. The results suggest that younger adults seemed to be quite good in fully adapting to their new body constraints. However, the adaptation process seemed to be reflected in changed judgment tendencies. Young adults applied an ideal to liberal judgment criterion for normal body properties, and only demonstrated rather conservative responses when judging their splinted hand (at both time points, directly as well as after 24h), reflected by both lower Hit and lower False-Alarm rates. The following two points might explain this brief change of heart: First, a process that we call here the criterion-instability-hypothesis. It would be conceivable that a habituation period of 24 hours was a phase of instability during which a new criterion needs to be built. In this case, the provided adaptation time may not have been sufficient to complete the build of a stable novel criterion. Participants might experience heightened ambiguity during the ongoing criterion formation process and choose a rather cautious approach. Interestingly, young adults appear to directly switch back to normal. This suggests that they are able to retrieve their previous experience-based criterion right after typical conditions are reinstated and that their prior criterion appeared not to be overwritten. At least this seemed to apply for the here tested timeframe. Secondly, the tendency to make more conservative (and not liberal) judgments for altered body properties might be routed in the anticipated consequences of incorrect judgments. It could be argued that the current task type elicits conservative judgments since judgment failure is anticipated with serious consequences.in the current study, a layer of insecurity is added by the introduced bodily alteration (e.g. enlarged hand due to a splint) and related changes in capabilities. In other words, when someone is not familiar with his or her body constraints, he or she acts more cautious compared to performing actions when body properties were normal and familiar.

Older adults were not as quick in adapting to altered body properties. In this group bodily alterations had a negative impact on judgment behavior in all dependent variables: judgment accuracy, perceptual sensitivity and judgment tendency. The older adults’ initially conservative judgment tendency for normal body properties was enhanced after putting on the hand splint. Interestingly, body alterations did not only influence judgments for the splinted hand but also for the non-splinted hand. In addition, the size estimation ability was closely related to the applied judgment tendencies. Older adults’ ability to estimate the size of their splinted hand was further correlated with accuracy and signal detection values. Thus, when body capabilities were altered, it seems as if older adults increasingly rely on perceptual abilities (i.e. estimation of size) when judging their action opportunities. The intensified reliance on perceptual abilities has also been emphasized in findings of an earlier study demonstrating that brain damage to the left ventro-dorsal stream seemed to disrupt the retrieval and processing of the learned criterion. Left hemisphere stroke patients’ ability to discriminate between possible and impossible actions seemed to be strongly associated with intact on-line body-perceptive processes [44]. Thus, we propose that a common underlying behavioural mechanism comes to the fore when being confronted with uncertainty or lost knowledge, namely the reliance on size perception.

Due to the fact that judgments for both hands seemed to be affected by bodily alterations we here assume that splinting of one hand may have led to a rather general interruption of body estimates, or going along with our hypothesis above, it may have led to a criterion-instability generalizing to both hands. While older adults’ judgment performances tended to improve after wearing the hand splint for 24 hours, the adaptation effect was not significant. This could be a result of a generally slower and less flexible adaptation process in older adults corresponding to what has been described to be an age-related decline of motor reaction capacity and speed [45, 46] as well as cognitive function [26, 47]. However, this also indicates, that older adults can, albeit more slowly, adapt to changing body constraints. This is confirmed by prior evidence, that older adults can for instance benefit from a feedback training that allowed participants to experience one hand within the opening [3]. In this previous study, training-induced improvements were present irrespective of whether the hand to be judged was the trained hand, thus a carry-over effect from the trained hand to the other hand was assumed.

Furthermore, older adults applied a highly conservative (or more cautious) judgment tendency for normal and altered body properties. Within the framework of the model of factors shaping judgment tendencies that has been described above, older adults may be more concerned about potential negative consequences of misjudgments and thus, less likely engage in perceived risky actions. The assumption that older adults are more cautious is confirmed by the error pattern in their judgment behavior reflecting an avoidance of False-Alarms and a lower rate of Hits (Fig 5). In addition, one may speculate that older adults are confronted with their declines on a daily basis. Particularly, under unknown conditions this may lead to instability of a criterion. This in turn elicits a rather conservative behavior.

## Conclusions

Due to the manifold circumstances that may lead to altered body constraints (e.g. accidents, bodily decline) and / or cognitive deficits particularly in older age (e.g. due to neurological disorders such as after stroke), the need for specifically designed diagnostic and training approaches becomes apparent. The current study examined judgment behaviour for normal vs. altered body properties in young and older adults. It has been demonstrated that younger adults are quite accurate in judging whether the hand fits into a horizontal opening.for normal (active hand: 81%, passive hand: 78%) as well as altered body properties (active hand: 81%; passive hand: 74%). Younger adults seemed to form a new judgment criterion within 24 hours of habituation to their splinted hand. Older adults performed worse in adapting to this change. The alteration of body parts seems to have led to an interruption or instability of the older adults’ judgment criterion, resulting in enhancing their conservative judgment tendencies. Although both groups improved their judgments after the habituation time of 24h, they not fully adapt to the new bodily constraints within the given timeframe.

The deviation in response tendencies between normal and altered body properties could be explained by a heightened instability. During a phase of instability in which a new criterion has to be formed in response to bodily alterations (criterion-instability-hypothesis).

Future fundamental research should focus on the effects of alterations (e.g. attention captivating stimulus alterations) or conditions (e.g. introducing active movements for the splinted hand; manipulating vision/occlusion of the hands) and the underlying mechanisms (e.g. type of splint representation: is the splint represented as body property versus external object). In addition, application-oriented studies should shed further light on the underlying adaptation processes in affordance judgments as well as the potential to support such processes by for example specifically designed training approaches. This may be specifically relevant for subjects with actual body alterations. For example, many major accidents lead to serious injuries and persons affected must cope with substantial and lasting changes of body properties. This also includes patients, who suffered from neurological diseases (e.g. stroke, traumatic brain injury) introducing sudden or fast declines in cognitive or motor function (for example, limb apraxia [48–50]; visuo-spatial neglect [51–53] and hemiplegia [54, 55]).
